# Mitigating structural racism to reduce inequities in sepsis outcomes: a mixed methods, longitudinal intervention study

**DOI:** 10.1186/s12913-022-08331-5

**Published:** 2022-07-30

**Authors:** Erika L. Linnander, Adeola Ayedun, Dowin Boatright, Kupiri Ackerman-Barger, Timothy I. Morgenthaler, Natasha Ray, Brita Roy, Steven Simpson, Leslie A. Curry

**Affiliations:** 1 Department of Health Policy and Management, Yale School of Public Health, New Haven, USA; 2 Yale Global Health Leadership Initiative, Yale School of Public Health, New Haven, USA; 3 Department of Emergency Medicine, Yale School of Medicine, New Haven, USA; 4 New Haven Healthy Start, New Haven, USA; 5 Betty Irene Moore School of Nursing, University of California Davis Health, Sacramento, USA; 6 Division of Pulmonary and Critical Care Medicine, Mayo Clinic, Rochester, USA; 7 Department of Medicine, Yale School of Medicine, New Haven, USA; 8 Division of Pulmonary, Critical Care and Sleep Medicine, School of Medicine, University of Kansas, Kansas City, USA

**Keywords:** Sepsis, Structural Racism, Organizational Culture, Racial Disparities, Leadership, Intervention Studies, Health Systems

## Abstract

**Background:**

Sepsis affects 1.7 million patients in the US annually, is one of the leading causes of mortality, and is a major driver of US healthcare costs. African American/Black and LatinX populations experience higher rates of sepsis complications, deviations from standard care, and readmissions compared with Non-Hispanic White populations. Despite clear evidence of structural racism in sepsis care and outcomes, there are no prospective interventions to mitigate structural racism in sepsis care, nor are we aware of studies that report reductions in racial inequities in sepsis care as an outcome. Therefore, we will deliver and evaluate a coalition-based intervention to equip health systems and their surrounding communities to mitigate structural racism, driving measurable reductions in inequities in sepsis outcomes. This paper presents the theoretical foundation for the study, summarizes key elements of the intervention, and describes the methodology to evaluate the intervention.

**Methods:**

Our aims are to: (1) deliver a coalition-based leadership intervention in eight U.S. health systems and their surrounding communities; (2) evaluate the impact of the intervention on organizational culture using a longitudinal, convergent mixed methods approach, and (3) evaluate the impact of the intervention on reduction of racial inequities in three clinical outcomes: a) early identification (time to antibiotic), b) clinical management (in-hospital sepsis mortality) and c) standards-based follow up (same-hospital, all-cause sepsis readmissions) using interrupted time series analysis.

**Discussion:**

This study is aligned with calls to action by the NIH and the Sepsis Alliance to address inequities in sepsis care and outcomes. It is the first to intervene to mitigate effects of structural racism by developing the domains of organizational culture that are required for anti-racist action, with implications for inequities in complex health outcomes beyond sepsis.

**Supplementary Information:**

The online version contains supplementary material available at 10.1186/s12913-022-08331-5.

## Background

Sepsis (a life-threatening organ dysfunction caused by a dysregulated host response to infection)[1] affects almost 2 million patients in the US annually [1–4], is one of the leading causes of mortality (nearly 350,000/year) [2] and is a major driver of healthcare costs ($62 billion/year) [5, 6]. Substantial racial inequities in sepsis care and outcomes exist. African American/Black (AA/B) and LatinX populations experience higher rates of complications, deviations from standard care, and sepsis readmissions compared with Non-Hispanic White (NHW) populations [7]. These inequities are not attributable to differences in genetic susceptibility, clinical needs, or patient preferences [8]. Structural racism is defined as “a system in which public policies, institutional practices, cultural representations, and other norms work in various, often reinforcing ways to perpetuate racial group inequity.” [9] Structural racism compounds factors at the hospital and community levels to generate far poorer sepsis care and outcomes for AA/B and LatinX patients [8, 10, 11]. Racial inequities are observed at all stages in the sepsis care pathway, from primary prevention to care after discharge, stemming from the triple burden [12, 13] of increased incidence of sepsis, socioeconomic disadvantage, and lower quality care [8, 14–20]. Sepsis Alliance, the nation’s first and leading sepsis organization, has emphasized the urgency of addressing racial inequities in sepsis care, prioritizing this issue in its mission and issuing a national call to action [21].

Despite clear evidence of the role that structural racism plays in driving inequities in sepsis outcomes [8], there are no evidence-based, prospective interventions to mitigate structural racism in sepsis care, nor are we aware of studies that report reductions in racial inequities in sepsis care as an outcome. Current clinical quality improvement efforts in sepsis care focus largely on early detection and protocol-driven treatment with well-established care bundles [22]; however, none of these include racial inequities as an outcome [23]. Further, “intervention research has rarely emphasized reduction of SRD as a strategy to reduce disparities” [24], and we are aware of no studies that engage health systems and community partners to address structural racism as a driver of inequities in sepsis. Realizing the full potential of recent major advances in basic science that improve identification and treatment of sepsis [22] will require collective action across health systems and community institutions using adaptive leadership [25] to create anti-racist systems change. [26]

Therefore, we aim to adapt, deliver, and evaluate a 2.5-year, coalition-based leadership intervention in eight U.S. health systems and their communities to improve the domains of organizational culture that are required to mitigate structural racism in sepsis care. We will adapt a proven intervention [27] that will develop and support Guiding Coalitions as they work to mitigate structural racism as a driver of racial inequities in sepsis care in their local context. We hypothesize that the intervention will generate longitudinal improvement in the domains of organizational culture that are required to mitigate structural racism in sepsis care. Using a longitudinal, convergent mixed methods approach we will: 1) quantify change in domains of organizational culture using a novel survey adapted from our previously validated instrument [28] (an innovation in measurement of structural racism), and 2) describe the experience of change within each system, integrating quantitative and qualitative data to develop a comprehensive understanding of impact and the mechanisms by which impact may have occurred. We also hypothesize that hospitals that demonstrate improvements in organizational culture will also demonstrate greater reductions in racial inequities in three sepsis-related outcomes: a) early identification (time to antibiotic), b) in-hospital mortality, and c) 30-day, all-cause hospital readmission, using comparative interrupted times series (ITS) analysis. The purpose of this paper is to present the theoretical foundation for the study, summarize key elements of the intervention, and describe in detail the study methodology to evaluate the intervention.

### Theoretical foundation

#### Coalition-based organizational change

Our team’s prior seminal work (the Leadership Saves Lives/LSL study), a novel, evidence-based organizational culture change intervention that focused on 30-day risk-standardized mortality rates (RSMR) for acute myocardial infarction (AMI), provides a robust foundation for the proposed study [29]. We intervened in 10 US hospital systems drawn from the Mayo Clinic Care Network (MCCN), working for 2 years with Guiding Coalitions (e.g., a wide range of clinicians, administrators and others both within and outside of the hospital), to reduce RSMR for AMI through improving key domains of organizational culture (i.e., learning and problem solving, senior management support, psychological safety). The 6 hospitals that demonstrated substantial shifts in culture showed a significantly greater decrease in RSMR than the 4 hospitals that did not experience such culture changes (RSMR reduced by 1.07 percentage points and 0.23 percentage points, respectively; *P* = 0.027). The evidence generated through LSL demonstrates that a coalition-based leadership intervention can change organizational culture with impact on a major clinical outcome [27]. Mechanisms for change included collaborating across historical, political, and organizational boundaries [30–32]. The LSL intervention required close collaboration with organizations outside of the hospital; hospitals reported planning to ‘transfer LSL’ to other complex health outcomes such as heart failure and stroke, suggesting its broader applicability. In addition, our prior research has also shown that health system links with non-health community organizations are associated with improved readmission rates [33], and that efforts to generate social capital in surrounding geographies and invest in social supports such as housing and community engagement are tied to better outcomes for a number of complex health conditions [34–36]. We anticipate similar types of collaborations with health care and non-health care partners will be developed by Guiding Coalitions in the proposed study. Finally, our validated measure of hospital organizational culture [28] will be adapted to address the domains of organizational culture that are required to mitigate structural racism, another important methodological advancement of the proposed study.

#### Evidence of structural racism in sepsis care and outcomes

AA/B and LatinX populations have higher rates of complications leading to sepsis [37], deviations from standard sepsis care, higher in-hospital mortality, and higher all-cause and sepsis readmissions compared with NHW populations [7, 10]. Sepsis-related mortality for AA/B patients is nearly double that for NHWs across all age groups (rate ratio = 1.78) nationally [38]. AA/B patients are twice as likely to die from severe infections driven by antibiotic resistance as compared to NHWs; many of these are sepsis deaths [21]. Racial inequities are observed at all stages in the care pathway for patients at risk of sepsis. At the level of primary prevention, AA/B and LatinX people experience higher levels of chronic comorbidities, and lower rates of access to primary care [17, 19, 20], including vaccination (which reduces incidence of sepsis) [16]. At the level of care seeking, AA/B and LatinX patients have well-justified mistrust in engaging with the health care system [14], demonstrate lower awareness of sepsis [39], receive less timely and accurate triage [18, 40], and receive lower intensity use of diagnostics and treatment (e.g., less likely to receive ICU care, longer length of stay, less frequent referral for follow-up care). AA/B patients are more likely to experience poverty and lack of insurance [8], both of which are associated with poorer care and outcomes along the care continuum, although many of these inequities persist when controlling for insurance status [41–43]. Finally, AA/B patients experience implicit bias at the provider and health care system levels; however, currently no studies examine the role of implicit bias in sepsis care [44–46]. Sepsis Alliance, the nation’s first and leading sepsis organization, has emphasized the urgency of addressing racial inequities in sepsis care, prioritizing this issue in its mission and issuing a national call to action [21].

#### Linking from underlying racism to clinical outcomes

**Fig. 1:**
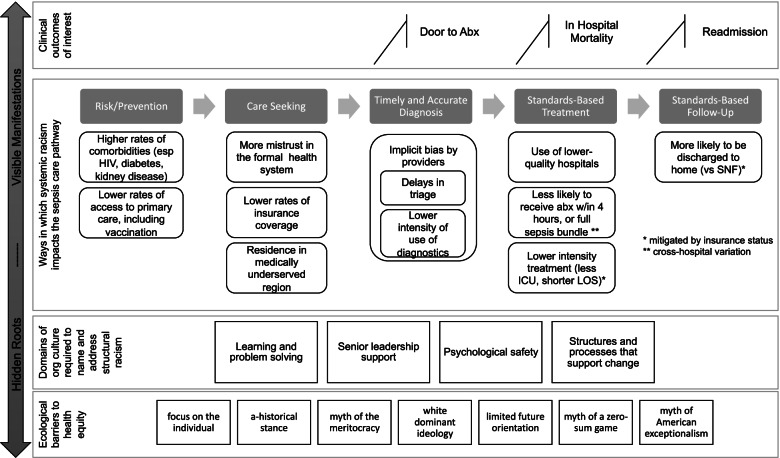
Conceptual model of how racism is manifest across the sepsis care continuum. This novel conceptual model below, developed based on synthesis of existing evidence on race-based inequities in healthcare and outcomes, shows how racism is manifest across the sepsis care continuum, and provides a starting point for our work to measure and intervene on the domains of organizational culture that are required to mitigate structural racism

The conceptual model below (Fig. 1), which we developed based on synthesis of existing evidence on race-based inequities in healthcare and outcomes, shows how racism is manifest across the sepsis care continuum, and provides a starting point for our work to measure and intervene on the domains of organizational culture that are required to mitigate structural racism [8, 14, 47–50]. At the foundational levels of the framework are ecological barriers to health equity, factors that are deeply embedded in our US society that perpetuate structural racism [51]. Through facilitated collaboration, Guiding Coalitions will increase their home systems’ attention to these factors. The next level defines domains of organizational culture associated with organizational capacity to mitigate structural racism. These domains, derived from our own work in organizational culture change and consistent with capacity for whole systems transformation, are the capacities that will be strengthened through the proposed intervention [27, 29, 30, 32]. The next level defines ways in which structural racism impacts the sepsis care pathway, moving from risk and primary prevention on the left to standards-based follow up care on the right. Participating sites will use their own data to name and prioritize the inequities most salient in their own systems and apply a proven strategic problem-solving process [52] to understand and address these symptoms of structural racism. Finally, at the highest level, we will measure the impact of these changes on racial inequities in care and outcomes for patients with sepsis.

## Methods/design

**Fig. 2:**
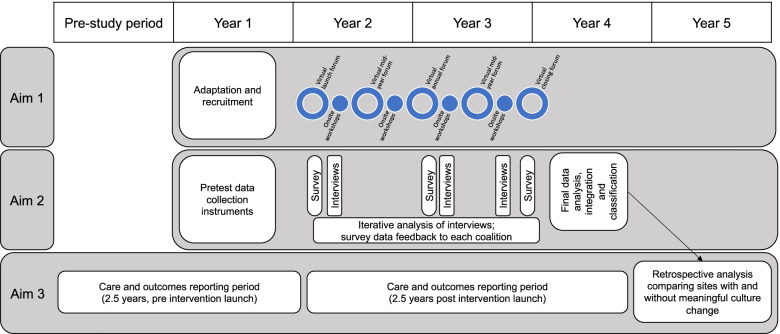
Study Timeline. We will employ a longitudinal mixed methods interventional study design that includes three aims: delivery of the intervention, evaluation of the impact of the intervention on organizational culture, and evaluation of the impact of the intervention on racial inequities in care and outcomes for patients with sepsis. The study is projected to take place over a 5-year project period

We will employ a robust, longitudinal mixed methods interventional study design that includes three aims: delivery of the intervention, evaluation of the impact of the intervention on organizational culture, and evaluation of the impact of the intervention on racial inequities in care and outcomes for patients with sepsis (Additional File 1: Supplemental Fig. 1). The study is projected to take place over a 5-year project period (Fig. 2). This description of the intervention and the associated evaluation has been prepared in alignment with the StaRI standards for the reporting of implementation studies (Additional File 2) [53].

### Site selection and intervention (Aim 1)

*Overview:* We will adapt and deliver a coalition-based leadership intervention in eight U.S. health systems and their surrounding communities to improve domains of organizational culture that are required to mitigate structural racism, with reducing racial inequities in sepsis care and outcomes as a shared goal. We will support these eight health systems to establish Guiding Coalitions made up of administrative and clinical leadership involved in sepsis care, patient advocacy representatives, and community organizations poised to address the impact of structural racism through collaboration. We will engage these coalitions in a 2.5 year, facilitated leadership intervention grounded in double-loop learning [27, 54, 55] to apply a strategic problem-solving method [52] to measure, understand, and address structural racism as a driver of racial inequities in sepsis care in their local context.

#### Sample

Health systems and their surrounding communities will be recruited from the membership of the MCCN, a group of independent health systems that purchase access to Mayo Clinic's tools and expertise, and that are vetted and selected for their commitment to high-quality patient experience and outcomes. These members represent all four census regions and all nine census divisions, serving sufficiently diverse patient populations to demonstrate the changes in racial inequities anticipated through the proposed work. We will use random sampling with a purposeful component [56] to select sites that include at least 5% proportion of AA/B clients and are diverse in geography and teaching status. Of MCCN’s 37 domestic (US) members, approximately 20 have a minimum patient population greater than 5% AA/B [average 12%]. We will work with each site to establish a Guiding Coalition [57] that represents the components of the system (within and beyond the health sector) required to address the impact of structural racism on sepsis care and outcomes (Table 1). Each site will be asked to name four core coalition members who will serve as senior champions for the project representing health systems administration, clinical leadership, and community engagement. These core members will participate in the cross-hospital forums and will lead the creation of the full coalition in their site, adapting guidance for coalition membership and structure to their unique context.

**Table 1:** Sample Coalition Membership. We will support eight sites to establish Guiding Coalitions made up of administrative and clinical leadership involved in sepsis care, patient advocacy representatives, and community organizations poised to address the impact of structural racism through collaboration

**Health systems leadership:**
• CEO or CMO *
• DEI officer
• Population health officer
**Clinical leadership:**
• Sepsis clinical champion: MD and RN *
• Emergency medicine
• Intensivist
• Hospitalist
• Primary care
**Support services:**
• Discharge planning
• Manager in charge of financial counseling/uncompensated care
• Post discharge rehab facilities (residential and OPT)
**Community networks:**
• Member of patient advisory board
• Community Services Administration *
• Existing and new community partners based on area of coalition focus

^*^ *Denotes core members of the coalition*

#### Intervention components

Based on our prior success in similar organizational change efforts [29], the 2.5 year intervention will include three components: 1) a series of five semiannual virtual, cross-site forums attended by four key members of each Guiding Coalition; 2) a series of four one-day workshops onsite with the full coalition at each hospital; and 3) a web-based platform to allow sites to share experiences and to serve as a repository for program resources. The content will build on and extend the previously published LSL curriculum [29]. *Semiannual forums* will bring sites together as a learning community for advice and problem solving on culture change to address structural racism in sepsis care. The sequence of forums will move sites from understanding and ‘buying in’ to the evidence base to sharing implementation challenges and successes. In the annual and closing forums, sites will vote for the “STAR” (Striving to Achieve the Remarkable) site that best exemplifies commitment to the objectives of the learning community, a highly motivational activity in LSL. *On site workshops* will build leadership capacity within the full Guiding Coalition to mitigate structural racism as it manifests in sepsis care. Each of the four workshops will include one full day of content, scheduled based on site preferences. The core curriculum for the workshops will incude both *how to work* (improving the domains of organizational culture that are required to mitigate structural racism and the underlying ecological barriers to health equity) and *what to work on* (strategic problem solving [52] to address root causes of inequities in sepsis care and outcomes in their site, see Additional File 1; Supplemental Fig. 2 for examples). In the first workshop, we will orient Guiding Coalition members to the intervention and the evidence base, promote reflection on their own measures of inequities in sepsis care and baseline organizational culture (survey measure described below), and name a problem statement and objective on which the group will focus their root cause analysis process. We will also invest in effective working relationships and processes among coalition members, including representation and role clarity [58], decision-making [59], and accountability [60]. Between workshops, the coalitions will be tasked with making and measuring progress toward addressing identified priority root causes of racial inequities in sepsis care in their home systems. At subsequent workshops, members will report on their progress, with the goal of solving implementation challenges and further developing their individual and group capacity for adaptive leadership [25, 26]. Additional workshop content will include diagnosing and shaping organizational culture, engaging conflict productively [61], using levels of analysis to diagnose organizational challenges [62, 63], and working with power and hierarchy [64, 65]. Content will be tailored to the local context by adjusting the timing of these modules to meet teams’ most pressing needs, adapting the specific examples and experiential learning exercises used in each module, articulating linkages between these content areas and ongoing work in each site, and encouraging each site to focus on root causes of inequities in sepsis care and treatment that are most salient in their environment. The facilitation team will include expert facilitators from across the United States, with backgrounds in organizational development, leadership education, anti-racist organizational change, and clinical quality improvement; most facilitators will be drawn from a faculty pool with deep experience developed through prior successful projects. Facilitators will work in teams of two or three, including a lead facilitator and additional experts tailored to the needs of each site. To promote standardization across sites while allowing for adaptation to local context, facilitators will use a fidelity checklist for each workshop. At the end of each workshop, the lead facilitator will prepare a structured written summary of decisions and action items for report back to the Guiding Coalition and to the full facilitation team. Between workshops, *a web-based information sharing platform* will serve two primary functions: (1) to serve as an accessible, up-to-date repository of project materials and references and (2) to support direct communication across hospital teams for sharing of successes, barriers, and project updates.

### Evaluation of impact on organizational culture (Aim 2)

**Fig. 3:**
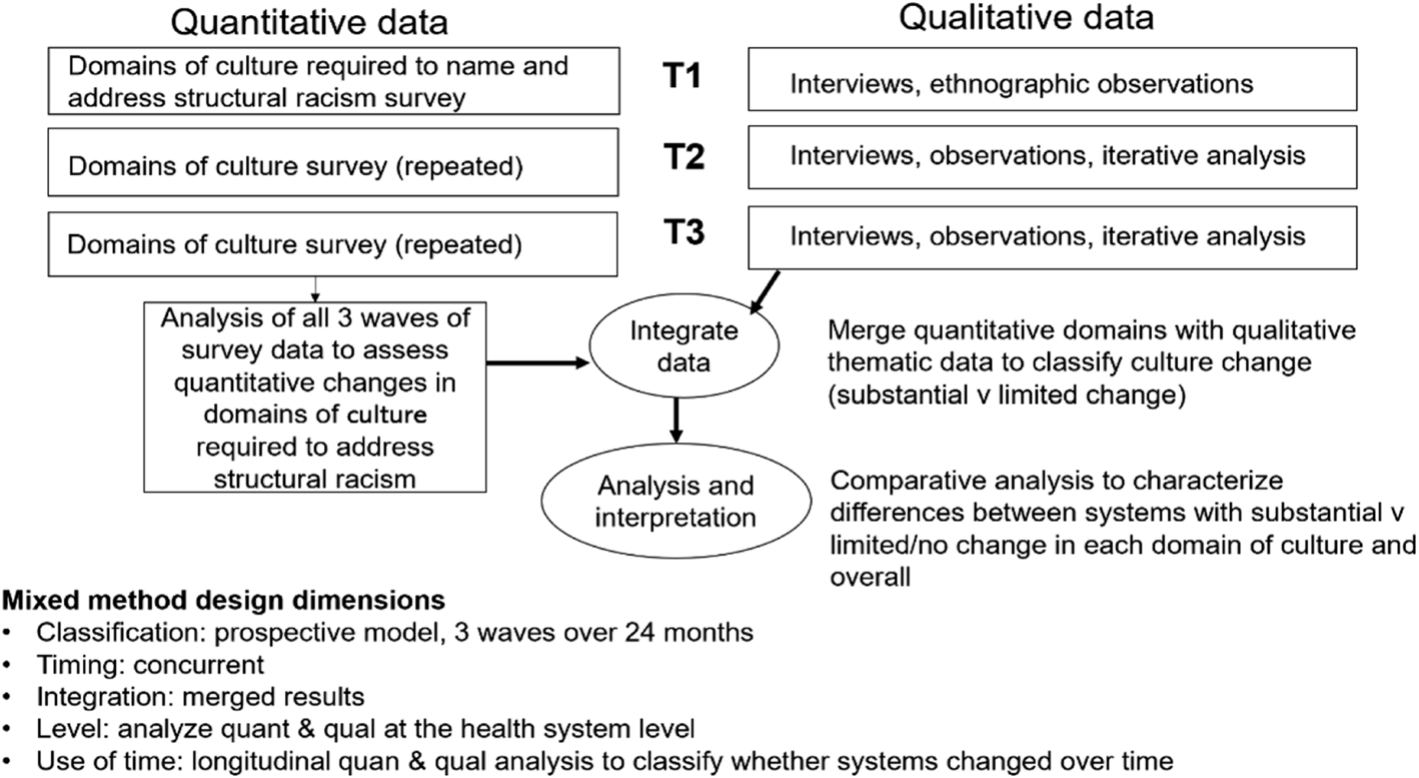
Convergent mixed methods design. As is recommended for evaluating complex interventions, we propose a convergent, longitudinal mixed methods design with: 1) a novel survey instrument (quant), 2) in-depth interviews (qual) and 3) ethnographic observations (qual), integrating quantitative and qualitative data at the analysis phase to develop a comprehensive understanding of intervention impact on organizational culture and mechanisms by which the impact may have occurred

The intervention will be evaluated using a robust, longitudinal mixed-methods design (additional file 1: supplemental Fig. 3). First, we will evaluate the impact of the intervention on the domains of organizational culture that are required to mitigate structural racism in care of patients with sepsis. As is recommended for evaluating complex interventions [66], we propose a convergent, longitudinal mixed methods design [67] (Fig. 3), with: 1) a novel survey instrument (quant), 2) in-depth interviews (qual) and 3) ethnographic observations (qual) [68], integrating quantitative and qualitative data at the analysis phase[69] to develop a *comprehensive understanding* of intervention impact on organizational culture and *mechanisms* by which the impact may have occurred.

#### Survey

All Guiding Coalition members at each system (*n* = 8 x ~ 15 members = 120) will be invited to participate in a survey using a novel instrument drawn from previously validated scales (Additional File 3) [28], which includes 30 items within 4 domains: learning and problem solving, senior leadership support, structures and processes that support change, and psychological safety. Example item: “*We have goals and metrics in place to guide our efforts to reduce racial inequities in care of patients with sepsis.*” Responses will be entered using the sliding bar function in Qualtrics, with anchors “never” to “always.” To ensure that our instrument captures new concepts related to anti-racist practice, we will conduct cognitive interviews [70, 71] (*n* = 8–10) with respondents similar to Guiding Coalition members, and revise our draft instrument as needed [28, 72–75]. Participants will complete the secure, web-based survey at the launch, midpoint and end of the intervention. To assess changes in 4 domains of organizational culture and overall, we will use hierarchical generalized linear modeling to estimate the association between mean scores and time, accounting for clustering of individual respondents within systems. We will use statistical analysis to determine significant changes at the level *p* < 0.05 for overall culture and each of the 4 domains for each system and the full sample. Analyses will be conducted using SAS V.9.4 and independently corroborated by two analysts. In addition to assessing the impact of the intervention in this study, the survey will be the first (to our knowledge) to measure aspects of organizational culture required for anti-racist systems change.

#### Interviews and observations

In-depth, in-person interviews will be conducted by trained members of the research team with different disciplinary backgrounds, using a standard interview guide (Additional File 4)**,** beginning with a grand tour question [76]: “*Tell me about your experiences in working to address racial inequities in sepsis care*,” with structured probes to encourage participants to describe specific examples or vignettes. The sample will include Guiding Coalition members and additional key informants [56] as needed to achieve thematic saturation [77] at each site. As is typical with in-depth interviewing [78], we anticipate interviews will be approximately 1 h in length. Interviews will be audiotaped and transcribed by independent, professional transcriptionists to enhance data reliability. At each site, we will also observe 2–8 h of daily routines in relevant community and hospital settings tailored to the sepsis project at each site.

#### Data collection

Observations will be completed by an experienced researcher using a standard observation template (Additional File 5) [79, 80] including context, stakeholders, and events, synthesized as field notes at the completion of the site visit. Intervention teams will participate in a debriefing session immediately upon return from site visits; reflections will be included in the qualitative data set for analysis. *Analysis:* Transcribed interview and ethnographic data will be analyzed by a 5-person multidisciplinary team using the constant comparative method of qualitative data analysis [81]. Coding of the data will include a series of iterative steps, beginning with an initial ‘start list’ of codes [82] to be refined during analysis of transcripts from subsequent interviews. We will conduct iterative coding and analysis with each wave of data collection, using negotiated consensus to resolve differences in interpretation, until a final code structure is established [82]. We will implement longitudinal analysis using matrices (rows = single case, group or theme, columns = time) to uncover patterns and interrelationships in change over time within and across health systems [83]. Data will be entered into ATLAS.ti to facilitate analysis. We will systematically search for disconfirming evidence [84], interview multiple participants in each health system for triangulation [85], and maintain a detailed audit trail to document analytic decisions [76]. To our knowledge, this will be one of the largest longitudinal qualitative data sets in the country [83], and could support sub-investigations on the nature of structural racism and health [31, 86].

Integration will be accomplished through merging in a mixed methods matrix [69, 87] at the final stage of analysis (Additional File: 1 Supplemental Fig. 4). For each site, we will integrate quantitative trend data (overall and by each domain of the survey) and qualitative data. We will classify each site as having positive or no change based on meeting either quantitative (statistically significant change in overall readiness score from baseline to follow up) or qualitative criteria (marked shift in culture characterized by substantial, consistent, specific illustrations of notable changes in three to four of the domains of culture and little to no disconfirming evidence). We will use comparative analysis to describe differences between the systems that achieve substantial change and those that do not. Finally, we will examine differences in clinical outcomes as described in Aim 3 below. Note: analysts will be blinded to all clinical outcomes data during this analysis.

### Evaluation of impact on racial inequities in sepsis care (Aim 3)

We will evaluate the impact of the intervention on racial inequities in three sepsis-related outcomes: a) early identification (time to antibiotic), b) in-hospital mortality, and c) 30-day hospital readmission, using comparative interrupted times series (ITS) analysis. *Time to Antibiotics*: We will use hospital EHR data to determine the difference in mean time from hospital arrival to first antibiotic administration for all AA/B patients and NHW patients and between LatinX and NHW patients respectively presenting with sepsis. This value will be calculated quarterly. We will use *t-tests* to measure statistically significant differences between AA/B and NHW patients, and LatinX and NHW patients. A clinically meaningful difference in mean time to antibiotic would be 30 min [88–93]. *In-Hospital Mortality:* We will use hospital EHR data to determine the difference in quarterly in-hospital mortality rate by 30 days for AA/B and NHW patients and LatinX and NHW patients admitted for sepsis. Rates will be standardized by age and sex [94]. At each time point, we will use *t-tests* to measure statistically significant differences between AA/B and NHW patients, and LatinX and NHW patients. Currently, the rates of in-hospital mortality for AA/B are nearly double those of NHWs, and although the overall rates of sepsis mortality have remained relatively stable over the past decade, this gap has persisted [38]. A clinically meaningful difference in rates of in-hospital mortality would be greater than 1%. *30-Day Hospital Readmission:* We will use hospital EHR data to determine the difference in quarterly 30-day, same-hospital, all-cause readmission rate for AA/B patients and NHW patients, and LatinX and NHW patients with an index hospitalization for sepsis. Rates will be standardized by age and sex. We will use *t-tests* to measure statistically significant difference between AA/B and NHW patients, and LatinX and NHW patients. A clinically meaningful difference in rates of 30-day readmission would be 5% [7]. *Sepsis Case Identification:* We will identify hospital admissions for sepsis using validated International Classification of Diseases, Ninth Revision, Clinical Modification (ICD-9-CM) diagnosis procedure codes for infection and organ failure. This strategy [4], known as the Dombrovskiy strategy, is more sensitive than methods that use only ICD-9-CM codes for sepsis [95] and will allow the investigative team to include patients with sepsis that may have otherwise been missed by chart review that would still be eligible for evidence-based practices. For each health system, we expect to be able to observe at least 140 AA/B patients with sepsis each year (within a sample of MCCN member health systems, average inpatient volumes are over 19,000, patient population is approximately 12% AA/B, and nationally, sepsis is present in 6% of adult hospitalizations) [4]; therefore, we anticipate being able to track enough cases within each health system for each clinical outcome at each quarter.

#### Analytic approach

We will conduct a comparative interrupted times series (ITS) analysis to examine the relation between the intervention and racial/ethnic differences in sepsis clinical outcomes between AA/B and NHW patients and between LatinX and NHWs. The analysis will be performed separately for our three key outcomes: 1) time to antibiotics, 2) in-hospital mortality, and 30-day readmission and stratified by response to the intervention (change/no change) (Additional File 1; Supplemental Fig. 5). We will consider a pre-exposure to the intervention time beginning 2.5 years (10 quarters) prior to starting the intervention and a postexposure period comprising 2.5 years (10 quarters) following the start of the intervention, with estimates made at every calendar quarter. ITS will be utilized to determine the difference in quarterly rates over time between changers (systems that responded to the intervention) and non-changers, health systems that did not respond to the intervention. Subsequently, ITS allows us to detect if an intervention has impact above and beyond secular trends when randomization is not a feasible option, and is ideal for evaluation of data collected across multiple equally spread time points by testing the changes in the slope of an outcome over time [96]. Additionally, ITS requires at least eight observations pre and post intervention [97]. We will utilize generalized mixed effect models in conjunction with segmented regression to evaluate the effectiveness of our intervention [98]. The model will control for patient-level characteristics, including age, sex, insurance status, Charlson Comorbidity index, and admission through the Emergency Department or transfer from another acute care facility, and hospital-level characteristics including size, academic vs community hospital, number of intensive care beds, annual sepsis case volume. We will also account for seasonality based on calendar quarter by including a “season” term alone and interacted with the treatment indicator and will assess for any residual autocorrelation using autoregressive integrated moving average (ARIMA). To allow a marginal interpretation of results, we will use a linear probability model for all analyses. Because outcomes of patients within a hospital are expected to be correlated, we will account for these non-standard variance–covariance structures by clustering at the hospital level. All coefficients will be modeled as fixed effects.

## Discussion

To our knowledge this is the first study to evaluate the impact of cultural change on racial inequities in care for patients with sepsis. Aligned with NIH and NIGMS priorities, the results of this study are expected to: 1) further the nascent scientific literature on conceptualizing and measuring structural racism and health outcomes, 2) generate community-driven innovations in sepsis care for dissemination through national organizations such as American College of Chest Physicians, and 3) provide much-needed evidence to inform local and national dialogue around structural racism and health. We fully recognize this study is an audacious proposition. It not only addresses a highly sensitive topic (structural racism), but it also seeks to make measurable change in the structural root causes of inequities in sepsis care. Importantly, we have balanced our ambitions for whole system change with careful operationalization of core constructs and detailed measurement approaches to understand the mechanisms of change. Further, the proposed study may be especially audacious in the context of COVID-19, which will likely strain organizational capacity to participate in change initiatives and is expected to change patient care processes and outcomes. As described above, a period of systematic stakeholder engagement will allow us to adapt the intervention for feasibility in the ever-changing context of COVID-19. Further, given the well-document impact of the pandemic in exposing structural racism and exacerbating racial inequities, the proposed study is both timely and urgent.

We have anticipated several potential limitations in the proposed methodology. First, to mitigate social desirability bias (participants reporting socially acceptable, rather than authentic, responses) [99], we will use established techniques including attention to our own positionality and engagement of a diverse research team, engagement of a wide range of key informants across the project, use of interview guides with scripted probes to elicit details that would be difficult to misrepresent, encouraging participants to share both positive and negative experiences, and cultivating longitudinal relationships to encourage candor [100–102]. Second, clinical outcomes data will be derived from the EHR, which is known to have high rates of missing or misclassification of socially assigned race. Although this is concerning, 1) evidence indicates that EHR-based clinical data provides more objective estimates than claims-based data for sepsis surveillance [4], 2) any bias is likely toward the null (as most errors are of identifying LatinX patients as NHW) [103–105] and 3) there is no alternative. Further, we anticipate that some sites may use this project as an opportunity to highlight and correct for historical underinvestment in systematic, reliable capture of socially assigned race and other important demographics in their EHR. Third, Coalition turnover is expected to occur, limiting paired analyses of quantitative data. However, our prior research found that neither coalition size nor turnover was associated with intervention outcomes [30] (an association that we will evaluate in the proposed study as well). Further, organizations that were more successful in driving changes in organizational culture were observed to proactively adjust their coalition membership to adapt to changes in organizational leadership, to engage emerging champions, and to account for the specific aspects of AMI care they were addressing. Additionally, our study is designed to measure systems-level (rather than individual-level) phenomena, and we anticipate that the roles represented on the coalitions will remain relatively stable even as individuals come and go. Fourth, as with all health interventions there is a lag in time between implementation of practice change and corresponding adjustments in outcome rates. Subsequently, we plan to track difference in AA/B and NHW patients and LatinX and NHW patients for each of our clinical outcomes for 2.5 years post intervention implementing different lag periods (up to 6 months of lag time between the end of the intervention and time of observed clinical outcomes). Fifth, we may face limitations in attributing observed changes to the intervention. Hospitals will be exposed to multiple sources of knowledge during the intervention and may also undergo substantial internal shifts in staff and priorities. The mixed methods design of the proposed study is expected to allow us to capture and understand factors beyond the intervention that may be influencing the results.

This project is expected to provide a solid foundation for natural extensions of future work to address racial inequities in health outcomes nationally, including: 1) adapting the intervention to address other clinical conditions for which structural racism is a major influence such as diabetes and heart disease, 2) if the intervention is successful, conducting implementation science studies to determine the necessary dose and core components of the intervention to inform efforts to scale up nationally, and 3) sustaining and enhancing our productive partnership with MCCN to serve as a platform for investigation of basic science and clinical innovations in sepsis. Finally, because this is practice-informed scholarship, we anticipate that systems will generate novel strategies and tools for clinical management of sepsis. This was the case in LSL, where we received two grants to support knowledge translation and packaging of tools for AMI care generated by the study [106]. There is a substantial opportunity to partner for practical dissemination of results and associated ‘toolkits’ through national organizations such as CHEST, Sepsis Alliance, and the Society of Critical Care Medicine.

Addressing the impact of structural racism on sepsis care will require urgent and courageous action across health systems and community institutions, supported by ways of working to collaborate effectively across historical, political, and organizational boundaries. Aligned with calls to action by the NIH and Sepsis Alliance to address inequities in sepsis care and outcomes, this paper outlines an ambitious and rigorous interventional mixed methods approach. It is the first study to intervene prospectively to mitigate effects of structural racism by developing the domains of organizational culture that are required for anti-racist action. The results of this study are expected to have implications for providers, policymakers, and other healthcare professionals committed to addressing systemic racism within and beyond care for patients with sepsis.

## Supplementary Information

**Additional file 1:** **Supplemental Figure 1. **Overview of study aims and sub-aims.** Supplemental Figure 2. **Examples of types of problems coalitions may prioritize. **Supplemental Figure 3. **Summary of evaluation approach, sampling, and analysis plans for each outcome. **Supplemental Figure 4. **Example of data integration to classify systems. **Supplemental Figure 5. **Example of trend analysis output.

**Additional file 2:** Completed StaRI checklist.

**Additional file 3:** Survey instrument.

**Additional file 4:** Interview guide.

**Additional file 5:** Observation guide.

## Data Availability

Not applicable—No datasets or materials have been generated or analyzed as part of this prospective description of the study protocol.

## Abbreviations

AA/B: African American/Black
AMI: Acute Myocardial Infarction
EHR: Electronic Health Record
ITS: Interrupted times series
LSL: Leadership Saves Lives
MCCN: Mayo Clinic Care Network
NHW: Non-Hispanic White
RSMR: Risk-Standardized Mortality Rate
