# Supplementary material for: Mitigating structural racism to reduce inequities in sepsis outcomes: a mixed methods, longitudinal intervention study
Source: BMC Health Serv Res. 2022 Jul 30;22:975. doi: 10.1186/s12913-022-08331-5 (PMC9338573; doi:10.1186/s12913-022-08331-5)

**Additional File 1: Supplemental Figures**

**Supplemental Figure 1. Overview of study aims and sub-aims**

**Supplemental Figure 2. Examples of types of problems coalitions may prioritize**

|  | Clinical outcome prioritized by the site | Problem (signal of systemic racism in sepsis care at that site) | Focal root cause (based on review of site-specific data) | Strategy (reflecting national best practice and local innovation) |
| --- | --- | --- | --- | --- |
| 1 | Inequities in time to antibiotics | Internal data on inequities in time to antibiotics are not considered valid or valued by thought leaders | EHR does not accurately capture socially assigned race | Strengthen internal processes for reliability and validity of data capture for socially assigned race |
| 2 | Inequities in time to antibiotics | LatinX patients with sepsis experience longer time to triage than their NHW counterparts | Implicit bias among providers results in errors in early identification of sepsis | Introduction of an electronic alert in the EHR |
| 3 | Inequities in readmission rates | AA/B patients receive less intensive follow-up services compared to their NHW counterparts | AA/B patients are more likely to receive multidisciplinary discharge huddle | Comprehensive dx plans provide linkage to supports for referral to home health services, nutrition, or supportive housing |
| 4 | Inequities in sepsis in-hospital mortality | AA/B patients are less likely to receive pneumococcal vaccination, controlling for insurance status | AA/B patients are less likely to have access to primary care | Cross-sectoral engagement campaign to increase primary care linkages in AA/B communities |
| The capacity of coalitions to name and address structural racism through each of these diverse projects will require positive shifts in organizational culture, including: learning and problem solving, senior leadership support, structures and processes that support change, and psychological safety. | | | | |

**Supplemental Figure 3. Summary of evaluation approach, sampling and analysis plans for each outcome**

| **Outcome** | **Design** | **Sample** | **Analysis** |
| --- | --- | --- | --- |
| **Aim 2:** Evaluate the impact of the intervention on the domains of organizational culture that are required to name and address structural racism to improve sepsis outcomes | Mixed methods, convergent design (qualitative = interviews and observations, quantitative=survey, integration= merged results) | For both interview and surveys, the sample is all members for Guiding Coalitions (n=10-20) in each health system (n=8), total n=80-160; 0, 18, 35 months | Qualitative = constant comparative method; quantitative = descriptive, time-trend analyses |
| **Aim 3:** Evaluate the impact of the intervention on racial inequities in early identification and clinical management of sepsis *(process of care)* | Quantitative measure: time to antibiotic | Deidentified patient-level data reflecting all sepsis admissions over a 5-year observation period | Interrupted time series |
| **Aim 3:** Evaluate the impact of the intervention on racial inequities in in-hospital mortality from sepsis *(clinical outcome)* | Quantitative measure: in hospital sepsis mortality rates | Deidentified patient-level data reflecting all sepsis admissions over a 5-year observation period | Interrupted time series |
| **Aim 3:** Evaluate the impact of the intervention on racial inequities in readmission rates from sepsis *(clinical outcome)* | Quantitative measures: 30-day all-cause readmission rates | Deidentified patient-level data reflecting all sepsis admissions over a 5-year observation period | Interrupted time series |

**Supplemental Figure 4. Example of data integration to classify systems**

| **Site** | **Quantitative data**  **(survey domains)** | | | | | **Qualitative data (interviews, observations, debriefs)** | | | **Overall classification** |
| --- | --- | --- | --- | --- | --- | --- | --- | --- | --- |
|  | Overall culture | Learning and problem solving | Psychological safety | Senior leadership | Supporting structures | Interview | Observation | Debrief |  |
| A | significant improvement | no  change | significant improvement | trend improvement | no  change | positive change | positive change | positive change | positive change |
| B | no  change | trend improvement | no  change | trend improvement | no  change | no change | change | no change | no  change |
| C, etc |  |  |  |  |  |  |  |  |  |

**Supplemental Figure 5: Example of trend analysis output**


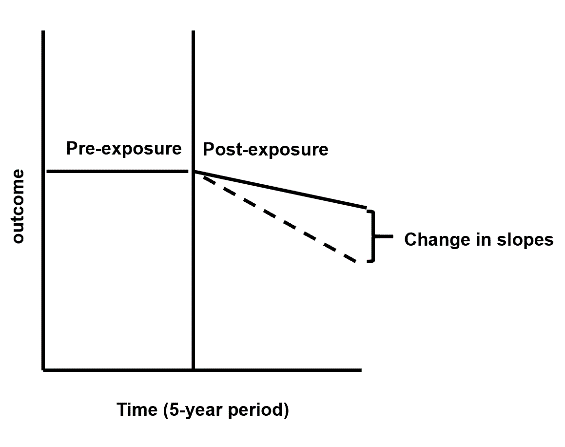

Supplement: Supplementary file 1 — Additional file 1: Supplemental Figure 1. Overview of study aims and sub-aims. Supplemental Figure 2. Examples of types of problems coalitions may prioritize. Supplemental Figure 3. Summary of evaluation approach, sampling, and analysis plans for each outcome. Supplemental Figure 4. Example of data integration to classify systems. Supplemental Figure 5. Example of trend analysis output. [file 12913_2022_8331_MOESM1_ESM.docx]
