# Supplementary material for: Mitigating structural racism to reduce inequities in sepsis outcomes: a mixed methods, longitudinal intervention study
Source: BMC Health Serv Res. 2022 Jul 30;22:975. doi: 10.1186/s12913-022-08331-5 (PMC9338573; doi:10.1186/s12913-022-08331-5)
Supplement: Supplementary file 3 — Additional file 3: Survey instrument. [file 12913_2022_8331_MOESM3_ESM.docx]

**Survey instrument**

This survey is intended to assess the capacity of your system to identify and address structural racism in care of patients with sepsis. By system, we mean the different organizations that might influence patients with sepsis. All information you provide is completely confidential, electronically secure, and only accessible by researchers at the Yale University. The survey will take approximately 15 minutes to complete. We appreciate your time.

Think about the work you are doing to address racial inequities in health outcomes, and indicate to what extent each of the statements below reflects those experiences.

***Learning and problem solving***

1. In this system, diverse clinicians and staff are encouraged to use creative problem solving to address racial inequities in sepsis care.

Never ____________________________________________________Always

2. In this system, there is good coordination among the different organizations involved with the care of patients with sepsis (e.g., primary care, hospitals, rehabilitation facilities, social services).

3. In this system, clinicians and staff who care for patients with sepsis hold each other accountable for high quality, equitable care.

4. In this system, we rely on data to guide our improvement processes aimed at reducing racial inequities in sepsis care.

5. In this system, we have frequent interactions with outside organizations to acquire new knowledge on how to reduce racial inequities in sepsis care.

6. In this system, clinicians and staff are interested in better ways of doing things.

7. In this system, clinicians and staff often resist new approaches.

8. In this system, clinicians and staff value new ideas.

9. Despite the workload, people in this system find time to review how the work is going.

10. In this system, someone makes sure that we stop to reflect on the team’s work process.

***Psychological safety***

1. If you make a mistake in this system, it is held against you.

2. People in this system are able to bring up problems and tough issues.

3. In this system, someone would deliberately act to undermine my efforts.

4. It is difficult to ask others in this system for help.

5. In this system, people’s unique skills and attributes are valued and utilized.

6. People in this system speak up to challenge assumptions.

***Senior leadership support***

1. Senior management has prioritized reducing racial inequities in care and outcomes for patients with sepsis.

2. Opinion leaders have indicated that racial inequities in care and outcomes for patients with sepsis can be improved.

3. Opinion leaders have encouraged changes in practices to reduce racial inequities in sepsis care.

4. We have adequate financial resources for personnel and equipment to reduce racial inequities in care of patients with sepsis.

***Structures and processes that support change***

1. We have a strategic plan to reduce racial inequities in care of patients with sepsis.

2. We have goals and metrics in place to guide our efforts to reduce racial inequities in care of patients with sepsis.

3. The system has legitimate processes in place to allow for the work of the guiding coalition to happen.

4. Stakeholders of diverse backgrounds, disciplines, and roles within our system have demonstrated buy-in for key elements of our work to reduce racial inequities in sepsis care.

5. Stakeholders of diverse backgrounds, disciplines, and roles within our system demonstrate appreciation of, and attention to, ways of working together.
