# Supplementary material for: Mitigating structural racism to reduce inequities in sepsis outcomes: a mixed methods, longitudinal intervention study
Source: BMC Health Serv Res. 2022 Jul 30;22:975. doi: 10.1186/s12913-022-08331-5 (PMC9338573; doi:10.1186/s12913-022-08331-5)
Supplement: Supplementary file 4 — Additional file 4: Interview guide. [file 12913_2022_8331_MOESM4_ESM.docx]

**Mitigating Structural Racism to Reduce Inequities in Sepsis Outcomes.**

**Draft Interview Guide**

**ESTABLISH TRUST**

**LISTEN INTENTLY**

**BE CURIOUS AND (APPROPRIATELY) INQUISITIVE**

**PURSUE UNANTICIPATED DIRECTIONS**

We are interested in understanding your organization’s experience with efforts to identify and mitigate structural racism as it shows up for patients with sepsis. We are very interested to learn about your successes, challenges, lessons learned and surprises along the way.

*We would like your permission to record this interview. First this lets us listen carefully to you rather than taking notes. Second, it means we will accurately capture our conversation for analysis. All information will be kept strictly confidential and no identifying information about you or your organization is included on the transcript. Digital files with audio recorded material will be deleted following transcription. If at any point you would like me to turn off the recorder, please let me know. You are free to decline to participate, to end our interview at any time for any reason, or to choose to skip any question. Choosing not to participate will not affect your relationship with the health system [hospital?].*

1. Please describe your current role.

a. What organization do you represent? What is your title? What are you responsible for?

Please also describe your role on the guiding coalition.

Alternate question for participants not on the guiding coalition: Please describe what you do in your organization and your role relative to the sepsis project.

**Potential probes:**

b. How long have you been in your role?

c. Who do you report to?

d. What other organizations or departments do you work closely with in your role?

*Aim: To establish rapport with the participant and provide a comfortable entry into the interview. Use this question to locate the person in the organization from his/her own perspective and gain a sense of their role in both the larger process of sepsis care and the structural racism and sepsis care guiding coalition.*

*Note for interviewer: If respondent appears to be unfamiliar with project/intervention, shift framing to efforts to improve quality of sepsis care or efforts to reduce sepsis mortality and readmission rates, especially among racial and ethnic minorities.*

2. Your hospital has been engaged in the project for [insert length of time]. Can you talk a little bit about how it has been going so far?

**Potential probes:**

a. What are some activities the coalition has engaged in?

b. Can you describe who has been involved?

c. Have there been any new or different approaches your team is engaging in since starting the project?

*Aim: To elicit descriptions of the coalition’s work to identify and address racial inequities in sepsis care and outcomes. Give the interviewer the*

*opportunity to explore a broad range of factors that the interviewee considers relevant to reducing race-based inequalititesin this setting.*

3. What aspects of the work have been going smoothly?

**Potential probes:**

a. Can you describe any processes established that have helped the work go smoothly?

*Aim: Identify elements of the environment that are facilitating efforts (structural, procedure, relational).*

4. We are interested in hearing more about setbacks or sources of resistance to the team’s efforts. Can you describe any bumps in the road so far? Any challenges that you think might lie ahead?

**Potential probes:**

a. How did your team respond to these challenges?

b. Has this challenge been resolved? If so, how? If not, can you describe why?

*Aim: Provide safe space to describe possible sources of tension or friction in the process of change. Uncover principles, strategies, and practices for managing change.*

5. Have there been surprises so far? Can you talk a little bit about those?

*Aim: Elicit descriptions of unanticipated elements in the environment, results, consequences that may be impacting the coalition’s work.*

6. Is there anything we haven’t talked about yet that you think would be important to help us understand how your coalition is addressing race-based inequities in sepsis care and outcomes?

*Aim: Close out the interview and allow the interviewee to reflect on any new concepts they consider relevant but not explored during the interview.*
