# Supplementary material for: Mitigating structural racism to reduce inequities in sepsis outcomes: a mixed methods, longitudinal intervention study
Source: BMC Health Serv Res. 2022 Jul 30;22:975. doi: 10.1186/s12913-022-08331-5 (PMC9338573; doi:10.1186/s12913-022-08331-5)
Supplement: Supplementary file 5 — Additional file 5: Observation guide. [file 12913_2022_8331_MOESM5_ESM.doc]

**Observation Guide**

**ESTABLISH TRUST**

**LISTEN INTENTLY**

**BE CURIOUS AND HUMBLE**

**OBSERVE IN THE BACKGROUND**

| **Selective observations (examples)** | **Length (mins)** |
| --- | --- |
| Hospital DEI or other committee meeting |  |
| Guided walk-around in CCU or other nursing unit |  |
| Guided walk-around in ED |  |

***Observations of rounding and meetings***

*We are interested in understanding how you provide care for patients with sepsis in this system. We would like to observe your team at work to learn how you do things here.*

| Physical Environment/ Context | - Where is the interaction taking place? - What is the space like?   - Energetic/Relaxed? Bright/Dim? Other?   - Who sits or stands where relative to others? |
| --- | --- |
| Actors/ Participants | - Who is doing what? - Who does not have a role? - What are the relationships between participants?   - Who interacts with whom?   - Who empowers or silences whom?   - How is power and authority exercised? - Who is missing? |
| Timing | - What is the frequency and duration of the meeting/rounds you are observing? - Is this a new or established process? |
| Informal factors | - What non-verbal cues do you observe? - What visual cues to you observe? - What symbols or symbolic acts do you observe? (e.g., procedures, artifacts of how the group works, how is the agenda set, ideas solicited) |

***Debrief notes***

Your observations will be used to inform interpretation of the interview data. At the end of each set of observations, please create a debrief note (approx 2 pages per hour of observation) that summarizes the experience with an emphasis on structures, processes and behaviors related to antiracist practice.
